# Iron supplementation in pregnant sicklers: an opinion

**DOI:** 10.1186/s12884-018-1894-y

**Published:** 2018-06-22

**Authors:** Desmond Aroke, Diego Nitcheu Tchouakam, Benjamin Momo Kadia, Simeon Pierre Choukem

**Affiliations:** 1 Fontem District Hospital, Fontem, Cameroon; 2 Health and Human Development (2HD) Research Group, Douala, Cameroon; 3 Roua District Hospital, Roua, Cameroon; 4 Foumbot District Hospital, Foumbot, Cameroon; 5 Faculty of Health Sciences, University of Buea, Buea, Cameroon; 6 Department of Internal Medicine, Douala General Hospital, Douala, Cameroon; 7 Faculty of Medicine and Pharmaceutical Sciences, University of Dschang, Dschang, Cameroon

**Keywords:** Sickle cell anaemia, Pregnancy, Iron supplementation

## Abstract

Morbidity associated with iron deficiency anaemia in pregnancy is increased in the presence of sickle cell anaemia. Iron supplementation in pregnant sicklers in a bid to resolve iron deficiency anaemia is recommended only after laboratory confirmation of iron deficiency. However, the greatest burden of sickle cell disease is seen in low and middle income countries where equipment for measuring body iron indices are unavailable.

Sickle cell anaemia is an inherited disorder of haemoglobin synthesis characterized by life-long severe haemolytic anaemia. People with sickle cell anaemia are at increased risk of iron overload from haemolysis and recurrent multiple transfusions. Iron overload a complication of sickle cell disease, which is more often in thalassemias, is typically given undue fear in sickle cell anaemia especially in patients with no recent transfusion history. About a third of the haemolysis in sickle cell anaemia is intravascular, and the resulting excess iron is lost in urine. This may lead to a negative iron balance and iron deficiency. There is little evidence of iron overload in pregnant sicklers, and iron deficiency may be more common than suspected. Even when iron overload does occur in a condition called siderosis, the deposited iron is irreversible and thus cannot be reused by the body in case of susceptibility to iron deficiency. More so, in pregnancy there is an increase in the body’s iron requirement by about 1000–1200 mg which is usually not met by dietary intake. Iron supplements could be given to pregnant sicklers, caution should however be taken in patients with history of recurrent transfusion.

Anaemia is a common and feared complication in pregnancy. The co-existence of iron deficiency anaemia and sickle cell anaemia worsens prognosis of pregnancy. Iron overload a possible complication of sickle cell anaemia is related to multiple transfusions. The urinary losses from intravascular haemolysis and increased dietary requirement in pregnancy predispose even pregnant sicklers to iron deficiency anaemia. Iron supplements should thus conveniently be given to pregnant sicklers with no history of recurrent transfusions.

## Background

Sickle cell anaemia (SCA) is the most common inherited condition worldwide. It is a disorder of haemoglobin synthesis characterized by sickled red blood cells with resultant life-long severe haemolytic anaemia, vaso-occlusion and complications secondary to ischaemia including pain crisis, organ damage, and a markedly shortened lifespan. About 2–3% of sub-Saharan Africans are born with the disease while the prevalence in USA stands at 1% [1]. Haemolytic anaemia is a major complication of SCA. Haemolysis leads to recurrent transfusions and thus risk of iron overload. Haemolysis in SCA is both intravascular and extra-vascular, and the former constitutes about a third of this haemolysis [2]. Urinary losses of iron from intravascular haemolysis may lead to negative iron balance and deficiency in iron. There is little evidence of iron overload in SCA, and iron deficiency may be more common than suspected [3, 4].

Anaemia is the most common medical complication of pregnancy and has potential for fatal consequences if not well managed [5]. The World Health Organization (WHO) has since 1998 recommended oral iron supplementation for pregnant women (60 mg of elemental iron and 400 μg of folic acid, once or twice daily) [6]. That notwithstanding, it is traditionally believed that iron deficiency is uncommon in sickle-cell disease (SCD) patients, and routine iron supplementation is not given. This view, is however being questioned by several authors, [7–9] especially in patients who have never or rarely been transfused. While some authors hold that iron supplementation should be provided to pregnant sicklers (PS) only in cases of documented iron deficiency [10], other authors have documented iron deficiency in both non-pregnant and PS [11–13]. They thus believe that providing supplementary iron to PS entails a negligible theoretical risk of iron overload for a substantial benefit, and thus propose routine iron supplementation in PS in the same manner as is given to other pregnant women.

Several studies have been carried out previously in a bit to assess iron stores in PS [11, 14–16]. Conflicting results obtained has led to varied opinions on the use of supplementary iron in PS. The differences in their outcomes were however thought to be related to their differences in methodology and study designs. Though the WHO currently has no recommendation for iron supplementation in PS, the traditional teaching has been that of no routine supplementation of iron in PS unless body iron indices confirm iron deficiency [17, 18]. However, most of the patients with this disease live in low/middle income countries, where health facilities are not equipped with the necessary tools to measure body iron indices. This paper provides an opinion on routine supplementation of iron in PS in such settings.

## Discussion

### Normal iron metabolism

The body’s iron content is maintained by the strained control of absorption based on body needs. About 10% of iron in diet is absorbed daily, mainly in the duodenum, and equal amounts are excreted through faeces, sweat, skin desquamation and urine [19]. Normally, iron deficiency increases iron absorption, while excesses decrease absorption [20].

### Iron metabolism in normal pregnancy

The normal body iron of adult females ranges from 2000 to 2500 mg which is much lower than the 3000 to 4000 mg found in adult males [21]. The normal iron requirement of pregnancy is approximately 1000 to 1200 mg [22, 23]. Of this, the foetus and placenta uses about 300 mg and about 200 mg is excreted through various routes, primarily the gastrointestinal tract [23]. These losses are mandatory and occur even in iron deficient states. The mother’s circulating erythrocyte increases by about 450 mL during pregnancy thus requiring another 500 mg of iron [5, 24]. Practically most of this iron is used in the second half of pregnancy. Thus, the iron requirement during the second half of pregnancy increases significantly, averaging 6–7 mg/day [5, 25]. This additional requirement is not available from body stores in most women. Hence, the necessary increase in mother’s red cell volume and haemoglobin mass fails unless exogenous iron is sufficiently provided [24]. When exogenous iron is not supplemented, the haemoglobin to maternal blood volume ratio falls appreciably. However, haemoglobin production remains unimpaired in the foetus because the placenta takes iron from the mother even in states of severe iron deficiency [26]. The dietary iron and iron mobilized from stores, is usually insufficient to meet the maternal iron needs imposed by pregnancy. Thus supplemental iron is essential even in the non-anaemic pregnant woman to prevent serum iron and ferritin concentrations from falling during the later half of pregnancy [5, 26].

### Iron excretion

The daily body requirements of iron vary depending on the person’s socio-demographic and physiological status [21]. Though iron is not lost conventionally, about 1 mg is excreted daily through varied mechanisms including sloughing of skin cells and shedding of cells lining the gastrointestinal and urinary tracts [21]. Few red blood cells are excreted in urine and feces as well. Most humans strictly preserve iron by recycling iron from old erythrocytes. In adult males, iron loss is negligible and usually compensated for by absorbing about 1 mg of iron daily [27]. On the contrary about 20 mg of iron is required daily for erythropoiesis. This conservation is paramount as many human diets contain iron just enough to replace the minimal losses. However, in women the blood lost in each menstrual cycle drains on average 30 mg of iron, so women in their reproductive years need to absorb much more than males [21]. Additionally in the presence of disease conditions with intravascular haemolysis, more iron is lost in urine in the form of haemoglobinuria [28].

### Pregnancy in sicklers

Studies demonstrate that pregnancy is not contraindicated for women with SCD [29]. However, antenatal management of women with SCD should be multidisciplinary. A management team should comprise of at least a haematologist, nutritionist and obstetrician. The team shares task to accomplish best possible outcome for the PS and her child [29, 30].

The antenatal clinic visit provides a forum for adequate counselling and planning of care for the PS and her baby for the antepartum and postpartum period [17]. The main objectives of the visit are usually to identify maternal risks for premature delivery, small for gestational age babies and risks for genetic foetal malformations [30, 31]. Antenatal assessment thereafter is similar to normal pregnancy [31].

PS with a normally growing foetus should be proposed elective birth through induction of labour, or by elective caesarean section if indicated, after 38 + 0 weeks of gestation due to increased risk of placenta insufficiency from multiple infarctions [30]. SCA should not in itself be considered a contraindication to attempting vaginal delivery or trial of scar. Counselling on different suitable positions for vagina delivery should be given to women who have had hip replacement surgeries secondary to avascular necrosis [30]. With these most PS usually opt for caesarean delivery and thus increased blood loss during delivery with resultant loss of body iron.

### Transfusion during pregnancy

Iron overload in sicklers and PS is mainly as a result of recurrent transfusions [2, 13, 32]. The relevance of prophylactic transfusions in PS is polemic. A retrospective study concluded that prophylactic transfusions, if started in the middle of pregnancy (20 weeks), may be of help in PS [33]. However others have concluded that regular transfusions from the start of pregnancy has no effect on the foetal and maternal outcome and is thus not necessary [34, 35].

A more cautious approach may be to avoid routine prophylactic transfusions for uneventful pregnancies and consider transfusing women with complications such as increased frequency of vaso-occlusive crisis, hypertension and severe anaemia. [36]. On the other hand, in women with previous abortions or with multiple gestations early use of transfusions may be beneficial to maintain a haemoglobin level of at least 9 g/dL [36] .

Another feasible approach for transfusions aimed at reducing the percentage of sickle haemoglobin (HbS), in PS who are not anaemic is exchange transfusion (replacing 500 mL of whole blood with 2 units of packed red blood cells). In low income settings, this procedure is performed manually to decrease the percentage of HbS to 30–40% of the entire Hb concentration and maintain a post-transfusion Hb of 10–11 g/dL [31]. Prophylactic transfusions in PSs should thus be avoided and exchange transfusion considered in patients with non- anaemic indications of transfusion.

### Iron overload in sicklers

**Fig. 1:**
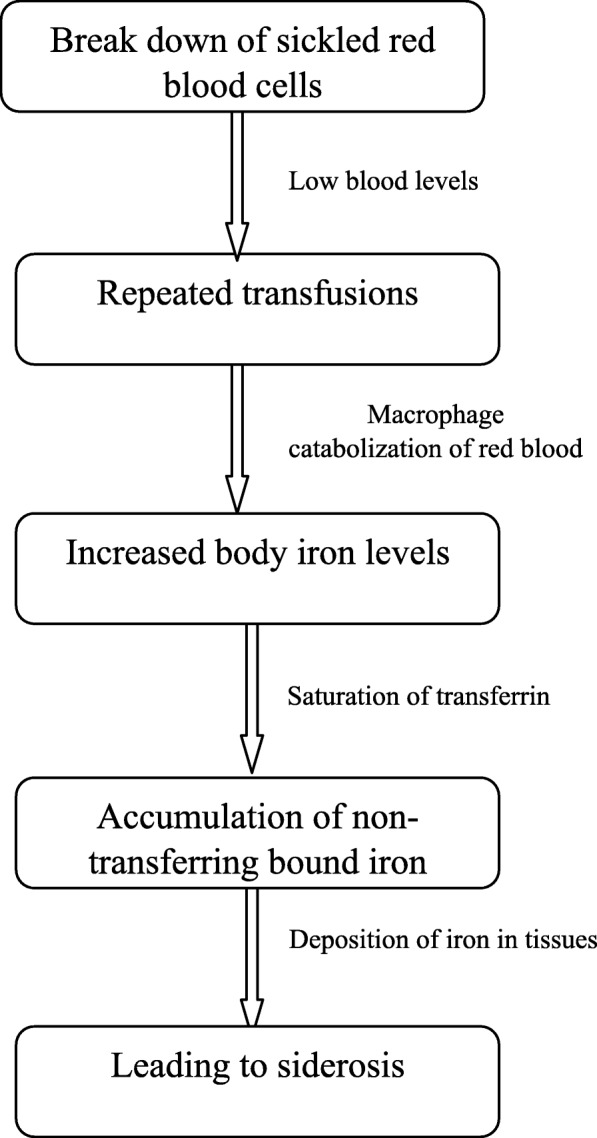
Mechanism of iron overload in sickle cell anaemia

Iron overload in patients with SCD is often undetected or not treated. In contrast to thalassemia patients, most patients with SCA are iron overloaded because of intermittent transfusions throughout their lives (Fig. 1). There is no evidence that SCD patients are spared the fatal consequences of iron overload.

There is no simple test to determine iron overload. Liver biopsy for quantification of liver iron is the gold standard for the diagnosis of hepatic iron overload [37]. Some schools recommend liver biopsies at the start of chelation and monitoring 2 yearly thereafter. Other non-invasive methods of quantifying liver iron are, the superconducting quantum interference device (SQUID), magnetic resonance imaging and computed tomography, but their clinical use is unproven [38]. Measurement of serial serum ferritins may help but can be unreliable because ferritin is an acute phase reactant and values are altered by liver disease, inflammation, vitamin C stores and even a painful crises [39, 40]. Serum ferritin used in many studies to evaluate iron status has thus led to an overestimation of iron overload and underestimation of iron deficiency [4, 41].

### Iron deficiency in pregnant Sicklers

The gold standard for the diagnosis of iron deficiency remains the absence of stainable iron in smears of bone marrow aspirate. However serum ferritin < 30 ng/mL though only 32% sensitive is currently considered diagnostic of iron deficiency [4]. The serum ferritin level and the total iron binding capacity are best used as complementary tests in the diagnosis of iron deficiency [41]. The free erythrocyte protoporphyrin (FEP) level has limited value in the diagnosis of iron deficiency because of non-specific elevation due to the high FEP content of reticulocytes [2]. Yet these indices cannot be readily measured in most health facilities in low income countries.

Anaemia is the most common medical complication of pregnancy and iron deficiency anaemia remains the most common cause of anaemia in pregnancy affecting 51–63% of pregnancies in Africa [42]. Anaemia in pregnancy is not merely common in Africa, it is also frequently severe and can be detrimental to both the mother and the foetus [5]. Maternal consequences of anaemia in pregnancy vary from reduced peripartal blood reserves, reduced physical activity and mental performance, cardiovascular strain, increased risk of peri-partum transfusion to maternal death while foetal consequences of low iron include; intrauterine growth restriction, hydrops foetalis, prematurity, low birth weight and death in utero [5]. These dreaded complications are well recognized and feared even by the WHO. In a bit to avoid these complications by preventing anaemia in pregnancy, WHO has since 1998 and currently still recommends iron and folic acid supplementation in pregnancy [6]. Current WHO recommendations states that for countries with prevalence of anaemia in pregnancy > 40% such as seen in African countries, daily 60 mg elemental iron +400μg of folic acid daily for 6 months during pregnancy and 3 months postpartum [26].

**Fig. 2:**
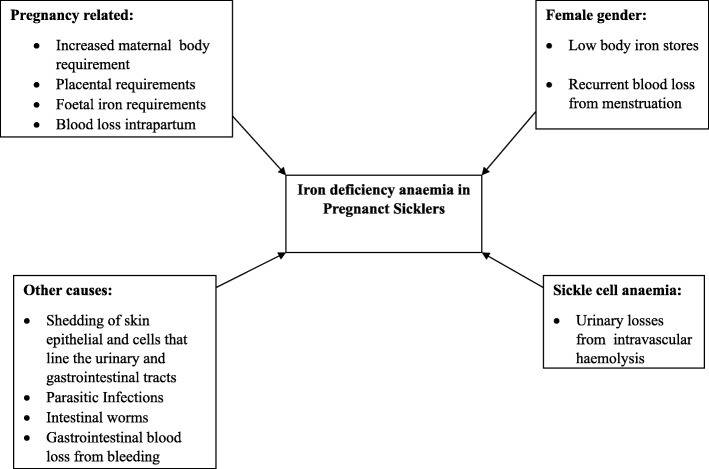
Causes of iron deficiency anaemia in pregnant sicklers

In PS the risk of iron deficiency anaemia as in other pregnancies still stands [11]. (Fig. 2). Sickle cell anaemia in pregnancy is itself burdensome as it is a high risk pregnancy. Chronic anaemia seen in these patients, increases the risk of maternal and foetal morbidity and mortality. Concurrent iron deficiency anaemia and SCA in pregnancy will surely only worsen the outcome of pregnancy.

In the past and recently much work has been done to assess the iron stores in pregnant women with sickle-cell disease, [11, 14–16, 43] with conflicting results and varied recommendations on the use of supplementary iron in these pregnancies.

Using serum iron and ferritin levels Abudu and Ya showed iron supplementation to be redundant in PSs [15, 43]. Abudu compared 20 pregnant HbAA women with 15 pregnant HbSS women at similar gestational ages, and found significantly higher values of serum ferritin in women with haemoglobinopathy [15]. Ya in his report comparing 100 non PS and 15 PS had serum ferritin levels in the normal and above normal ranges for both groups [43].

In other reports with about the same sample sizes and using both serum ferritin and bone marrow stainable iron as means of diagnosis of iron deficiency, Oluboyede, Anderson and Roopnarinesingh demonstrated the need for iron supplementation in PSs [11, 14, 16]. Anderson and Roopnarinesingh after controlling for bleeding, infections and infestations, showed no stainable iron in the bone marrow of 50 and 80% of the PSs respectively [14, 16]. Oluboyede assessed 22 PS and 18 non PS, and found significantly lower transferrin saturation in the pregnant than non pregnant population. 63% of the PS and 50% of the non PS had scanty or no bone marrow iron stores [11]. This strongly suggests iron deficiency is a likely occurrence in sicklers and more so in PS.

### Iron supplementation in pregnancy: Opinion

Iron overload is a true but rare complication of SCA. It is more often seen in the thalassemia variants of SCD than SCA. Chronic and acute haemolysis in SCA necessitates recurrent transfusions and thus iron overload [44]. However about a third of this haemolysis is intravascular and leads to iron loss in urine.

Though iron overload usually follows recurrent transfusion, the serum ferritin concentrations in patients who have received < 5 units of transfusion are generally not much different from patients who have never been transfused [44, 45]. Also studies show that transfusion related iron overload requiring chelation is only after 20–30 units of red blood cell transfusions in an adult [41]. Even so, the recent use of exchange transfusion in SCA patients with non-anaemic indications for transfusion has further reduced the risk of iron overload. Additionally, in patients with long term history of iron overload (with renal and splenic siderosis), the iron deposits are irreversible and thus even these patients are prone to iron deficiency anaemia [46]. In pregnancy the body’s total iron requirement surpasses the daily nutritional intake and thus predisposes to iron deficiency anaemia [24, 26].

In people with SCA it is recommended to test body iron indices before the decision to supplement iron in pregnancy is made [17, 18]. However, in sub-Saharan Africa with the greatest disease burden these machines are not readily available. That notwithstanding, microcytosis which is usually characterised by a low mean cell volume or mean cell haemoglobin usually provides a clue for either of the thalassemias or iron deficiency anaemia [13, 28]. However thalassemias are uncommon in sub-Saharan Africa, microcytosis could thus comfortably be used as a clue for iron deficiency anaemia in PS [28].

## Conclusion

One may argue for the need to avoid iron supplements in PS in order to prevent iron overload. Indeed, people with SCA are at risk of iron overload, but this usually follows multiple transfusions indicated for haemolytic crisis. Nevertheless, a third of the haemolysis in SCA is intravascular and leads to urinary iron loss. The realisation that iron deficiency anaemia in SCA may be more common than reported and that the risk of iron deficiency anaemia even increases in PS, may suggest that routine supplementation of iron in PS is necessary. Exchange transfusion rather than normal transfusion should be encouraged in non-anaemic indications of transfusion. In case of antecedents of multiple transfusions, iron supplements should be routinely provided, with microcytosis being used as a clue for iron deficiency. In case of recent multiple transfusions, iron supplements should be avoided. Clients with no history of transfusions or with ≤5 units of blood transfusion should be routinely supplemented with iron in pregnancy. In PS with 6 to 20 units of blood transfusions, microcytosis should be used as a clue to suspect iron deficiency while iron supplements should best be avoided in PS with greater than 20 units of blood transfusion. Future studies should focus on comparing iron stores in PS with and without history of transfusion, on measuring efficacy of microcytosis as a marker of iron deficiency anaemia in PS.

## Abbreviations

PS: Pregnant sicklers
SCA: Sickle cell anaemia
SCD: Sickle cell disease
WHO: World Health Organization
